# EXAMINING HUMAN-ANIMAL INTERACTIONS AND THEIR EFFECT ON FRAILTY IN LATER LIFE: A SCOPING REVIEW

**DOI:** 10.1093/geroni/igac059.3029

**Published:** 2022-12-20

**Authors:** Ashley Taeckens-Seabaugh, Mary Corcoran, Kevin Morris

**Affiliations:** University of Denver, Denver, Colorado, United States; University of Denver, Denver, Colorado, United States; University of Denver, Denver, Colorado, United States

## Abstract

Research suggests that human-animal interactions (HAIs) can improve the health and well-being of humans throughout their lifespan. While HAIs may facilitate healthy aging broadly, scant research has focused on HAIs as an intervention for adults aged 50 and older as it pertains to a comprehensive perspective of frailty. Moreover, scholarly literature lacks a consistent frailty definition, resulting in a lack of cohesion when evaluating the effectiveness of frailty interventions. This scoping review research proposes a comprehensive frailty definition and explores what is known about HAI interventions available to older adults as they relate to frailty statuses. Despite broad inclusion criteria, only four articles were relevant to this literature review, confirming the scarcity of relevant completed research thus far. Thematic analysis of reported results includes dog ownership as a protective factor regarding frailty statuses, the interconnected health effects of pet ownership, and meaning and purpose implications. Future interdisciplinary research should consider HAIs outside of pet ownership as frailty interventions for older adults, be mindful of population differences as they relate to intervention effectiveness, and work towards a universal, comprehensive definition of frailty that will aid in evaluating the frailty intervention effectiveness.

